# Quality Assessment of Private Practitioners in Rural Wardha, Maharashtra

**DOI:** 10.4103/0970-0218.39241

**Published:** 2008-01

**Authors:** Enakshi Ganguly, PR Deshmukh, BS Garg

**Affiliations:** Department of Community Medicine, Mahatma Gandhi Institute of Medical Sciences, Sewagram, Wardha - 442 102, Maharashtra, India

**Keywords:** Quality assessment, quality assurance, private practitioners, healthcare services, health infrastructure

## Abstract

**Objective:**

To assess the quality of care provided by private practitioners in rural areas of Wardha district.

**Methodology:**

The study was carried out in three primary health centres of Wardha district. 20% of the 44 registered private practitioners were selected randomly for the study. The data was collected using checklist through direct observation for the infrastructure. Assessment of quality of services delivered, 10 consecutive patients were observed and also the medical practitioner was interviewed. Supplies and logistics were assessed through observation.

**Results:**

All the facilities were sheltered from weather conditions and 90% had adequate waiting space. But, drinking water and adequate IEC material was available in only 20% facilities. Complete history taking and relevant physical examination was done in only 20% cases. Only 20% practitioners recorded blood pressure and 30% recorded temperature in cases with fever. Provisional diagnosis was not written in any of the case and only 20% explained prescription to the patients.

**Conclusion:**

There is considerable scope to improve the quality of services of private practitioners. To achieve this quality assurance programs may be initiated along with the training of private medical practitioners.

## Introduction

General private practitioner is the most sought out healthcare provider as has been confirmed by number of studies.(1) The services of the private sector are perceived to be far better than the public sector in most developing as well as developed nations.(2) India has to step forward to strengthen the primary healthcare system, and integration of services of the private practitioners along with the government health services has been recommended as a possible approach to increase the coverage of primary healthcare in India.(3) But before this movement proceeds, quality is to be assured at each step, so that services of the private practitioners continue to meet demands and requirements of the consumers within limited costs. Hence, the present study was undertaken to assess the quality of care in rural areas of Wardha district, Maharashtra.

## Material and Methods

The study was carried out in three primary health centres (PHC) of Wardha district, namely Anji, Gaul and Talegaon, which are the field practice areas of Mahatma Gandhi Institute of Medical Sciences (MGIMS), Sewagram after obtaining permission from the Institutional Ethical Committee.

A list of 44 (Anji - 14, Gaul - 8, Talegaon - 22) registered private medical practitioners in the above three PHC areas was prepared. Twenty percent of them, i.e. 10 in total, were selected using simple random sampling from each PHC area. Thus, two MBBS doctors (Bachelor of Medicine and Bachelor of Surgery), three BHMS doctors (Bachelor of Ayurvedic Medicine and Surgery), four BAMS doctors (Bachelor of Homeopathic Medicine and Surgery) and one BUMS doctor (Bachelor of Unani Medicine and Surgery) were studied.

After obtaining the consent, data were collected on infrastructure by the investigator, through direct observation of the facilities. For assessment of quality of services delivered, 10 consecutive patients were observed after obtaining consent for each private practitioner while they were being attended at the facility. The practitioner helped to recruit the patient. Parameters specific to the patient were assessed by actually following the patient flow through the clinic session, and also by interviewing the private medical practitioners using structured interview schedule. Supplies and logistics were assessed through direct observation. Data on maintenance of records were collected by review of records.

For all the above observations, a predesigned and pretested checklist was used, which was adopted from Primary Health Care Management Advancement Programme (PHC-MAP) module for Assessing the Quality of Service. The data, thus collected, were analyzed using epi_info 6.0.

## Results

### Quality of infrastructure and maintenance

A total of 10 private practitioners were studied. It was observed that adequate space for waiting was available in 90% clinics. All such places were sheltered from adverse weather conditions such as excessive sunlight, rain and winds. Drinking water for the waiting patients or clients was available in only 10% clinics. Eighty percent of the clinics had power supply round the clock or a backup system. All the clinics had examination table. General cleanliness of the clinics was overall satisfactory. When a finger was run across the corners for dust on benches or examination table, cleanliness was found to be acceptable in more than 90% of time. A provision for a screen to ensure privacy during examination was available in 80% clinics. The availability of basic equipment (stethoscope and sphygmomanometer) was reasonably good (70%) in the clinics. However, a weighing machine was missing or not functional in 80% of the centres. The availability and display of IEC materials to impart health education was grossly poor (80%) [Table 1]. Antiseptic solution for hand washing was not available in any of the clinics.

**Table 1:** Infrastructure facilities and maintenance at the private clinics

Infrastructure facilities at the private clinic	Number (*n* = 10)	Percentage
Adequate waiting space	9	90
Sheltered from weather conditions	10	100
Drinking water available	2	20
Power supply available	6	60
General cleanliness at facility	4	40
Screen for examination	8	80
IEC materials displayed adequately	2	20

### Quality of services

The average consulting time at these private clinics was 4.3 ± 0.7 h/day. The average number of patients at these clinics was 34 ± 9.3/day.

During the assessment, we studied 35 patients with respiratory illnesses, 15 of cardiovascular disorders and 20 with abdominal complaints. The remaining 30 patients had general health complaints. In our assessment of services provided, we found that almost all the patients were asked about their chief complaints, but only 20% providers inquired the history of present and past illnesses. A complete history-taking and relevant physical examination was done only in 20% cases. Vital parameters like pulse were read in only 40% cases; only 20% practitioners checked blood pressure; temperature was recorded only in 30% cases with fever. Moreover, other parameters like counting respiratory rate, examining the eyes for pallor or jaundice or palpation of lymph nodes, etc. were grossly neglected. Systemic examination, where relevant, was conducted in 30% of cases. The respiratory system was assessed for most cases up to 80%. Provisional diagnosis was not written in any of the cases. Only 20% private practitioners explained the prescription to their patients [Table 2].

**Table 2:** Quality of services provided by the private practitioners

Professional competence of provider	Number	Percentage
Asked chief complaints (*n* = 100)	100	100
Asked present history (*n* = 100)	20	20
Asked history of past illness (*n* = 100)	10	10
Asked complete history (*n* = 100)	0	0
Pulse palpated (*n* = 100)	40	40
BP measured (*n* = 100)	20	20
RR counted (*n* = 100)	0	0
Temperature recorded (*n* = 100)	30	30
Eyes examined for pallor or	10	10
jaundice (*n* = 100)		
Lymph nodes (*n* = 100)	0	0
Auscultation of RS (*n* = 35)	28	80
Auscultation of CVS (*n* = 15)	3	20
Performed per abdominal examination (*n* = 20)	6	30
Interpersonal qualities of the providers (*n* = 10)	
Explained prescription to patient	2	20
Provide health education	3	30
Provide nutritional counselling	1	10
Confirmed whether patient	1	10
understood instructions Discuss if patient had any questions	0	0
Refer for specialist care	2	20

None of these private practitioners practised hand washing after examination of the patient. Few clients (30%) were given health education and still fewer (20%) were given basic nutritional education. Moreover, the patient's understanding of the instructions was given importance in only 10% cases. Nowhere the client was encouraged to ask questions.

Referral services were also weak. Only 20% of the practitioners guided the patients to the proper referral centres if required. These referrals were mostly made for the purpose of higher investigations. None of the patients was given appointment for next visit [Table 2].

### Maintenance of records

Review of records reveals poor maintenance of records. Only 40% of private practitioners maintained a minimal record of their patients, which included the name, age of the patient and the amount due to be paid for the visit or prescribed medicines from the clinic.

## Discussion

There is a huge gap between the expected quality of outpatient care and the quality of care that is delivered in developing countries. Provider performance is a function of competence (professional and interpersonal) and infrastructural facilities. The present study has revealed several lacunae in private healthcare in rural Wardha, which has been strength of other studies on the subject.

### Infrastructure

In the present study, the waiting space for the patients was found to be adequate with power supply round the clock and satisfactory cleanliness. Many studies point to the higher utilization of private healthcare services due to availability of these basic infrastructural attributes in different parts of the country. The utilization of private sector was found to be high (69.05%) in two districts of Madhya Pradesh.(4) Another study reported the cleanliness of the hospitals in private sector to be 80%.(5)

### Services

In the present study, professional competence of the private practitioners was found to be low. Privacy during examination was satisfactorily maintained. Rohde and Vishwanathan(3) found that no physical examination was conducted in 47% cases as compared to more than 85% in the present study. They further found that the practitioners refer critical cases to government centres, which is consistent with our findings. Among other causes of referrals, the most common was found to be for laboratory investigations. Attempt to study the interpersonal communication of providers in terms of explaining the prescriptions and provisional diagnosis, providing health education and relevant nutritional counselling, confirmation of patient's understanding of instructions and encouraging patients to ask questions, etc., showed poor results in more than 80% cases. Lack of communication between provider and patients lead to inadequate gathering of information during consultation, leading to incorrect diagnosis, inadequate treatment and explanation, and inadequate understanding on part of client and thus, inadequate follow-up of instructions.(6)

Other attributes studied were handwashing practices and the availability of antiseptic solutions for handwashing. This was not found in the present study. Various other studies point to good handwashing practices either with soap or antiseptic solutions, but they are limited to urban clinics.(7)

### Records

Maintenance of records was found in only 40% of clinics. These records did not contain any information regarding the diagnosis, detailed case management or follow-up. As such no studies on maintenance of records in the private sector in rural areas could be found.

## Conclusion

There is a considerable scope for the improvement of basic facilities, service delivery and maintenance of records. To achieve this, there is an imperative need to equip the health providers with training and reorientation to patients' needs and also strengthening the counselling skills. Quality assurance programmes may be established in which such a study is repeated at regular intervals and feedback given to the private practitioners; based on which they could improve the quality of care on continuous basis. Such initiatives might strengthen the public-private partnerships.
